# Supplementary material for: Interlaboratory Comparison Study on Ribodepleted Total RNA High-Throughput Sequencing for Plant Virus Diagnostics and Bioinformatic Competence
Source: Pathogens. 2021 Sep 12;10(9):1174. doi: 10.3390/pathogens10091174 (PMC8469820; doi:10.3390/pathogens10091174)
Supplement: Supplementary file 1 [file pathogens-10-01174-s001.zip › pathogens-1306912-Supplementary File S1.pdf]

## General Remarks:

Make sure that all liquids are centrifuged briefly before use as the enzymes etc may stick to the lid due to transportation. We could only deliver the exact volumes needed for the RiboMinus kit due to the high costs.

## Step 1: Sample preparation

Before you start your extraction, please combine the freeze-dried content of vials TPS1 to TPS6 (= Sample 1) and the contents of vials TPS7 to TPS12 (= Sample 2) in an extraction bag (Bioreba). Continue immediately with RNA extraction.

## Step 2: RNA extraction using the RNeasy Plant Mini Kit (Qiagen)

Per sample provided:
3,5 ml  6 M Guanidine hydrochloride extraction buffer
0.5 ml ethanol ≥96%
1 x Grinding bag
1x QIAshredder spin column (lilac)
1x RNeasy spin column
3 x 1.5 ml collection tube
4 x 2 ml collection tube
1 ml RPE buffer
700 µl RW1 buffer
50 µl RNase free water

To be provided by the participant:
Micro centrifuge
Hand held homogenizer or Homex 6
Thermomixer or heatblock

1. Add 3,5 ml GH extraction buffer (6M guanidine hydrochloride, 0.2 M sodium acetate pH 5.2, 25 mM EDTA and 2.5% PVP-10) to each sample bag (sample 1 and sample 2,

Bioreba bag) and homogenise the tissues using for instance a Homex or hand held homogenizer (Bioreba).

2. Bring 1 ml of that plant extract to a 1.5 ml collection tube.
3. Incubate samples for 10 min at 65 °C and mix every 1 min.
4. Centrifuge for 2 min at 16,000 x g
5. Transfer 500 µl supernatant to a QIAshredder spin column (lilac) placed in a 2 ml collection tube, and centrifuge for 2 min at full speed.
6. Pipet 450 µl of flow-through to a new 1.5 ml collection tube
7. Add 225 µl ethanol (96–100%) and mix immediately by pipetting. Do not centrifuge and proceed immediately to step 8.
8. Transfer the sample, including any precipitate that may have formed, to a RNeasy spin column (pink) placed in a 2 ml collection tube.
9. Close the lid gently, and centrifuge for 15 s at ≥8000 x g
10. Put the column in a new 2 ml collection tube
11. Add 700 µl Buffer RW1 to the RNeasy spin column
12. Close the lid gently, and centrifuge for 15 s at ≥ 8000 x g to wash the spin column membrane
13. Put the column in a new 2 ml collection tube
14. Add 500 µl Buffer RPE to the RNeasy spin column.
15. Close the lid gently, and centrifuge for 15 s at ≥ 8000 x g to wash the spin column membrane
16. Put the column in a new 2 ml collection tube
17. Add 500 µl Buffer RPE to the RNeasy spin column.
18. Close the lid gently, and centrifuge for 2 min at ≥ 8000 x g to wash the spin column membrane.
19. Put the column in a new 2 ml collection tube and centrifuge for 1 min at full speed.
20. Place the RNeasy spin column in a new 1.5 ml collection tube.
21. Add 50 µl RNase-free water directly to the spin column membrane.
22. Close the lid gently, and centrifuge for 1 min at ≥8000 x g (≥10,000 rpm) to elute the RNA

Store the RNA extract at –20 °C for short term storage (up to 3 months), and at -80 °C for long term storage (> 3 months).

# Step 3: Ribodepletion using RiboMinus™ Plant Kit for RNA-Seq

For detailed information, see: https://assets.thermofisher.com/TFS-Assets/LSG/manuals/ribominus_plant_man.pdf

### Materials Needed

- Total RNA (see below; from the last step)
- RiboMinus™ Plant Kit for RNA-Seq (RiboMinus Magnetic Beads, RiboMinus Plant Probe, Hybridization Buffer and DEPC-treated water will be provided)
- Magnetic separator (Not provided)
- Sterile, RNase-free microcentrifuge tubes and water baths or heat blocks set to 70–75°C and 37°C (Not provided)
- **Glycogen, 20 µg/µL (Cat. no. 10814-010 Invitrogen). Attention: This item needs to be stored -5°C to -30°C, although approved for shipment on wet ice, we cannot influence and guarantee speedy delivery times we therefore leave it up to you to decide whether you continue with the aliquot provided or order fresh glycogen from Invitrogen!**
- 3 M sodium acetate (no pH specified) in RNAse-free water (provided)
- 96–100% cold ethanol and 70% cold ethanol (provided)

### Hybridization Step

Instructions are provided below to perform hybridization for 1–10 µg of your total RNA samples with the RiboMinus™ Plant Probe. To process >10 µg total RNA sample, divide your sample into two subsamples samples, each containing <10 µg total RNA but only continue with ONE subsamples as you will be provided with chemicals to process only a total of 2 samples (Sample 1 and Sample 2) but not anymore subsamples.

1. Set a water bath or heat block to 70–75°C.
2. To a sterile, RNase-free 1.5 mL microcentrifuge tube, add the following and mix by pipetting:
    - Total RNA (1–10 µg): <10 µL
    - RiboMinus™ Probe (15 pmol/µL): 10 µL
    - Hybridization Buffer: 100 µL
3. Incubate the tube at 70–75°C for 5 minutes to denature RNA.
4. Allow the sample to cool to 37°C slowly over a period of 30 minutes by placing the tube in a 37°C water bath (a heat block can be used if the temperature can gradually be cooled down see information below). To promote sequence-specific hybridization, it is important to allow slow cooling. **Do not cool samples quickly by placing the tubes in cold water, for example.**

While the sample is cooling down, proceed to Preparing Beads.

**Preparing Beads**

1. Resuspend RiboMinus™ Magnetic Beads thorough vortexing.
2. Pipet 750 µL of the bead suspension into a sterile, RNase-free, 1.5 mL microcentrifuge tube.
3. Place the tube with the bead suspension on a **magnetic separator** for 1 minute. The beads settle to the tube side that faces the magnet. Gently aspirate and discard the supernatant.
4. Add 750 µL sterile, DEPC Water to the beads and resuspend beads by slow vortexing.
5. Place tube on a magnetic separator for 1 minute. Aspirate and discard the supernatant.

6. Repeat Steps 4–5 once.
7. Resuspend beads in 750 µL Hybridization Buffer and transfer 250 µl beads to a new tube and maintain the tube at 37ºC for use at a later step.
8. Place the tube with 500 µL beads on a magnetic separator for 1 minute. Aspirate and discard the supernatant.
9. Resuspend beads in 200 µL Hybridization Buffer and keep the beads at 37°C until use.

**Removing rRNA**

1. After the 37°C incubation step of the hybridized sample (above), briefly centrifuge the tube to collect the sample to the bottom of the tube.
2. Transfer the sample (~120 µL) to the prepared RiboMinus™ Magnetic beads from **Step 9** (Preparing Beads, above). Mix well by pipetting up and down or low speed vortexing.
3. Incubate the tube at 37°C for 15 minutes. During incubation, gently mix the contents occasionally. Briefly centrifuge the tube to collect the sample to the bottom of the tube.
4. Place the tube on a magnetic separator for 1 minute to pellet the rRNA-probe complex. Do not discard the supernatant. The supernatant contains RiboMinus™ RNA.
5. Place the tube with 250 µL beads from **Step 7** (Preparing Beads, above) on a magnetic separator for 1 minute. Aspirate and decant the supernatant.
6. To this tube of beads, add ~320 µL supernatant containing RiboMinus™ RNA from Step 4, above. Mix well by pipetting up and down or low speed vortexing.

7. Incubate the tube at 37°C for 15 minutes. During incubation, gently mix the contents occasionally. Briefly centrifuge the tube to collect the sample to the bottom of the tube.
8. Place the tube on a magnetic separator for 1 minute to pellet the rRNA-probe complex. Do not discard the supernatant. The supernatant contains RiboMinus™ RNA.
9. Transfer the supernatant (~ 320 µL) containing RiboMinus™ RNA to a new tube.

**Concentrating RiboMinus™ RNA Using Ethanol Precipitation**

To ensure recovery of smaller (<200 nt) RNA, concentrate the RiboMinus™ RNA using ethanol precipitation as described below. Transfer the RiboMinus™ RNA sample into a clean, RNAse-free 1.5 mL or 2 mL microcentrifuge tube.

1. Add the following components to RiboMinus™ RNA:
    - 1 µL glycogen (20 µg/µL)
    - 30 µL of 3 M sodium acetate
    - 750 µL of 100% ethanol
2. Mix well and incubate at –80°C for a minimum of 30 minutes.
3. Centrifuge the tube for 15 minutes ≥12,000 x g at 4 °C. Carefully, discard the supernatant without disturbing the pellet.
4. Add 500 µL 70% cold ethanol.
5. Centrifuge the tube for 5 minutes at ≥12,000 x g at 4 °C. Discard the supernatant without disturbing the pellet.
6. Repeat Steps 4–5 once.
7. Air-dry the pellet for ~5 minutes. Resuspend the RiboMinus™ RNA pellet in 30 µL DEPC-treated water.

8. Place RiboMinus™ RNA on ice and proceed to desired downstream application or store RiboMinus™ RNA at –80 °C.

## Step: Sequencing preparation

You will receive details on sample preparation by Eurofins Genomics. Two barcodes for labelling your samples are included should you not have received any by Eurofins. They need to be transferred to your Eurofins Genomics account, so please send the email address you registered with to Heiko for transferral.
